# Local and non-local chemical potential and hardness: a grand canonical ensemble approach

**DOI:** 10.1007/s00894-025-06311-0

**Published:** 2025-02-18

**Authors:** Paulino Zerón, Maurizio A. Pantoja-Hernández, Marco Franco-Pérez, José L. Gázquez

**Affiliations:** 1 Departamento de Química, Universidad Autónoma Metropolitana-Iztapalapa, Av. San Rafael Atlixco 186, 09340 Mexico City, Mexico; 2 Facultad de Química, Cd. Universitaria, Universidad Nacional Autónoma de México, 04510 Mexico City, Mexico

**Keywords:** Conceptual density functional theory, Global, local, and non-local chemical potential and hardness, Fukui function, Fukui function kernel, Site and bond reactivity

## Abstract

**Context:**

The formulation of conceptual density functional theory in the grand canonical ensemble provides a theoretical framework that allows one to establish additional insights about the response functions that characterize this approach. In particular, through this procedure, one can establish the local counterpart of the chemical potential which, when integrated over all the space, leads to the global quantity and the local counterpart of the hardness that not only provides a function free of ambiguities, but also generates through its integration over all the space the well-defined value of the global quantity given by the difference of the vertical first ionization potential and electron affinity. In the present work, the non-local counterpart of these local reactivity descriptors is derived making use of the Fukui kernel descriptor previously developed by us. Then, the local and non-local chemical potential and hardness, thus obtained, are applied to study site and bond reactivities of several systems, to rationalize the behavior of kinetic and thermodynamic properties, through the chemical information that these indexes provide.

**Methods:**

The electronic structure calculations required to evaluate the reactivity indexes analyzed in this work were done with the PBE0 exchange–correlation energy functional. The geometry optimization was done in all cases in a modified version of the NWChem program, while the Hirshfeld population analysis was done in a modified version of the demon2k program. For the electrophilic addition of hydrogen halides (HX) to several substituted ethenes and the hydration reaction of aldehydes and ketones, the 6-311G** basis set was used, while for the bond enthalpies of chemical reactions where there is a homolytic bond break and the trans influence in which the lability of the leaving ligand is modified by the ligand opposite to it, the Def2-TZVP was used.

**Supplementary Information:**

The online version contains supplementary material available at 10.1007/s00894-025-06311-0.

## Introduction

Recently, the formulation of conceptual density functional theory (CDFT) in a temperature-dependent theoretical framework has been developed [1–5], by assuming that a molecule may be considered an open system that may exchange electrons with the reservoir (bath) in which it is immersed so that the number of electrons will fluctuate. This formulation implies that one needs to invoke the grand canonical potential to determine the response functions that provide information about the intrinsic chemical reactivity of a molecule and which are expressed, mainly, as derivatives of the average energy, $\langle E\rangle$, or of the average electronic density, $\langle \rho \left(\mathbf{r}\right)\rangle$, of the ensemble, with respect to the average number of electrons, $\langle N\rangle$.

An important element of this procedure is that the effect of the temperature is to smoothen the behavior of $\langle E\rangle$ and $\langle \rho \left(\mathbf{r}\right)\rangle$ with respect to $\langle N\rangle$ so that the derivatives to all orders exist and may be determined analytically. However, the values of the average energy, the average electronic density, and its first, second, and so on derivatives with respect to the average number of electrons, at temperatures of chemical interest, are basically equal to those determined in the limit when $T$ goes to zero. Additionally, the intrinsic chemical reactivity, which is described through the derivatives with respect to $\langle N\rangle$, must be evaluated, in principle, at $\langle N\rangle ={N}_{0}$, where ${N}_{0}$ is an integer that corresponds to the number of electrons of the ground state system being analyzed. Therefore, the derivatives with respect to $\langle N\rangle$ are determined first for the temperature-dependent expression, through the fractional charge $\omega$, defined as the difference between the average number of electrons and the ${N}_{0}$ electron system, $\omega =\langle N\rangle -{N}_{0}$, and the resulting equation is evaluated at $T=0$ and $\omega =0.$ Thus, all the equations concerning the reactivity indexes presented in this work are evaluated for these two values.

In this context, CDFT is based on four fundamental quantities. The first derivative of the energy with respect to the number of electrons, known as the chemical potential, was identified with the negative of the intuitive concept of electronegativity [6] through a generalization established by Iczkowski and Margrave [7] of the expression derived for this property by Mulliken [8]. In a grand canonical framework, the appropriate definition is given by $\mu ={\left(\partial \langle E\rangle /\partial \langle N\rangle \right)}_{v\left(\mathbf{r}\right)}$, which when evaluated with the three-ground-state ensemble model, composed of the species with ${N}_{0}-1$, ${N}_{0}$, and ${N}_{0}+1$ electrons in their corresponding ground states [9], one finds that the limit, when the temperature goes to zero and the fractional charge $\omega$ is equal to zero, becomes equal to [1, 10]

1 $$\mu =-\left(I+A\right)/2 ,$$

where $I$ is the vertical first ionization potential

2 $$I={E}_{{N}_{0}-1}-{E}_{{N}_{0}} ,$$

and $A$ is the vertical electron affinity

3 $$A={E}_{{N}_{0}}-{E}_{{N}_{0}+1} .$$

On the other hand, the second derivative of the energy with respect to the number of electrons was identified [11] with the intuitive concept of hardness developed by Pearson to establish the hard and soft acids and bases principle [12, 13], which for the temperature-dependent case would be given by $\eta ={\left({\partial }^{2}\langle E\rangle /\partial {\langle N\rangle }^{2}\right)}_{v\left(\mathbf{r}\right)}$, in the limit when the temperature goes to zero and $\omega$ is equal to zero, becomes equal to

4 $$\eta =\left(I-A\right)\delta \left(\omega \right) ,$$

where $\delta \left(\omega \right)$ is the Dirac delta function.

A relevant aspect of the relationship of the first and second derivatives of $\langle E\rangle$, with respect to $\langle N\rangle$ with the intuitive concepts of electronegativity and hardness, comes from the results expressed in Eqs. (1) and (4). The former, Eq. (1), indicates that the chemical potential is equal to the negative of the electronegativity of Mulliken which is known to follow the same tendencies observed in the qualitative scale developed by Pauling [14, 15], while the latter, Eq. (4), indicates that, if one eliminates the Dirac delta function, it leads to the expression $\eta =I-A$, which reproduces the tendencies of the qualitative scale developed by Pearson. An analysis of several definitions of hardness in the temperature-dependent framework [16, 17], shows that the quantity $\left(I-A\right)$ is indeed the one that characterizes this reactivity index, indicating that, in this context, the Dirac delta function may be ignored.

The next fundamental quantity is the first derivative of the electronic density with respect to the number of electrons, which is known as the Fukui function [18], which in the limit when the temperature goes to zero and $\omega$ is equal to zero becomes

5 $$f(\mathbf{r})=\left({f}^{-}(\mathbf{r})+{f}^{+}(\mathbf{r})\right)/2 ,$$

where

6 $${f}^{-}\left(\mathbf{r}\right)={\rho }_{{N}_{0}}\left(\mathbf{r}\right)-{\rho }_{{N}_{0}-1}\left(\mathbf{r}\right) ,$$

and

7 $${f}^{+}\left(\mathbf{r}\right)={\rho }_{{N}_{0}+1}\left(\mathbf{r}\right)-{\rho }_{{N}_{0}}\left(\mathbf{r}\right) ,$$

are the directional Fukui functions. The places where ${f}^{-}\left(\mathbf{r}\right)$ is large correspond to the best sites to donate charge, while the places where ${f}^{+}\left(\mathbf{r}\right)$ is large correspond to the best sites to accept charge [19]. Additionally, if one neglects the relaxation effects associated with the remotion or the addition of charge, ${f}^{-}\left(\mathbf{r}\right)$ and ${f}^{+}\left(\mathbf{r}\right)$ reduce to the density of the highest occupied (H) and lowest unoccupied (L) molecular orbitals, respectively [20]. This identification together with the rule $\Delta \mu$ big is good for chemical reactivity provides strong support to the frontier orbital theory.

Finally, the fourth fundamental quantity of CDFT corresponds to the second derivative of the electronic density with respect to the number of electrons, the dual descriptor [21, 22], which in the limit when $T\to 0$ and $\omega$ is equal to zero becomes equal to

8 $$\Delta f\left(\mathbf{r}\right)=\left({f}^{+}\left(\mathbf{r}\right)-{f}^{-}\left(\mathbf{r}\right)\right)\delta \left(\omega \right) .$$

As in the case of hardness, the relevant chemical information of the dual descriptor is contained in the quantity $\left({f}^{+}\left(\mathbf{r}\right)-{f}^{-}\left(\mathbf{r}\right)\right)$ so that one can ignore the Dirac delta function [23].

The importance of these four quantities to explain relevant aspects of the global reactivity of a molecule in the case of the chemical potential and the hardness and regioselectivity features in the case of the Fukui function and the dual descriptor lies, in part, in the fact that they are associated with intuitive concepts so that through them one translates the complex information contained in the molecular wavefunction into chemically meaningful quantities [24–32].

However, in order to obtain additional information contained in the global descriptors, one may define the associated local quantity to identify its spatial distribution that indicates the sites of the molecule that make the greatest contribution to the global value, and one may also define the associated non-local quantity that shows the spatial distribution of the local quantity and, therefore, the regions of the molecule that make the greatest contribution to the global value [33–36].

Recently, we presented a derivation of the local chemical potential, the local hardness, the chemical potential kernel, and the hardness kernel, with a methodology based on a zero-temperature CDFT description that makes use of the chain rule for functional derivatives [37]. The objective of the present work is to derive the expressions for these quantities using the grand canonical ensemble approach, to establish the similarities and differences between the two formulations, and to apply the new expressions to the analysis of site and bond chemical reactivities.

## Theoretical development

The detailed procedure to derive the local counterpart of the global chemical potential and hardness was given in Ref. [38], while the one corresponding to the Fukui kernel was done in Ref. [39]. Here, we present a summary of them, to derive through their combination the non-local counterparts of the chemical potential and the hardness.

The development of the local chemical potential [38] departed from the consideration that the average electron density may be considered as the local counterpart of the average electron number

9 $$\langle N\rangle =\int \langle \rho \left(\mathbf{r}\right)\rangle d\mathbf{r} .$$

Then, since the chemical potential may be expressed as the ratio of the thermal fluctuations between $\langle N\rangle$ and $\langle E\rangle$ and the thermal fluctuations of $\langle N\rangle$, that is

10 $$\mu =\frac{\langle NE\rangle -\langle N\rangle \langle E\rangle }{\langle NN\rangle -\langle N\rangle \langle N\rangle } ,$$

through Eq. (9), one can define a local chemical potential, namely

11 $$\mu \left(\mathbf{r}\right)=\frac{\langle \rho \left(\mathbf{r}\right)E\rangle -\langle \rho \left(\mathbf{r}\right)\rangle \langle E\rangle }{\langle NN\rangle -\langle N\rangle \langle N\rangle } ,$$

that, clearly, satisfies the relationship,

12 $$\mu =\int \mu \left(\mathbf{r}\right) d\mathbf{r}.$$

By making use of the three-ground-state ensemble, one can show that when the temperature goes to zero and $\omega$ is equal to zero, the local chemical potential becomes equal to

13 $$\mu \left(\mathbf{r}\right)=-\left(I{f}^{-}\left(\mathbf{r}\right)+A{f}^{+}\left(\mathbf{r}\right)\right)/2 .$$

This expression shows that the local chemical potential, which provides information on how the global property $\mu$ is distributed in the molecule, provides relevant information about the reactivity of the different sites. The quantity $-I{f}^{-}\left(\mathbf{r}\right)$ may be associated, following the arguments given in Ref. [40], with an average local ionization energy, which has been shown to provide information about the most reactive sites of a molecule for electrophilic attack [41–45], while the quantity $-A{f}^{+}\left(\mathbf{r}\right)$ has been used to describe the reactive sites for a nucleophilic attack [46–48]. On the other hand, the quantity that combines both, $-\left(I{f}^{-}\left(\mathbf{r}\right)+A{f}^{+}\left(\mathbf{r}\right)\right)/2$, is rather interesting in itself, because it is the local version of the electronegativity proposed by Mulliken.

Once the local chemical potential has been defined, the local hardness may be obtained through the expression:

14 $$\eta \left(\mathbf{r}\right)={\left(\frac{\partial \mu \left(\mathbf{r}\right)}{\partial \langle N\rangle }\right)}_{v\left(\mathbf{r}\right)} ,$$

which, in the limit when $T\to 0$ and $\omega$ is equal to zero, leads to

15 $$\eta \left(\mathbf{r}\right)=I{f}^{-}\left(\mathbf{r}\right)-A{f}^{+}\left(\mathbf{r}\right) .$$

A relevant aspect of this result is related to the fact that the integral of the local hardness over all the space is given by

16 $$\eta =\int \eta \left(\mathbf{r}\right) d\mathbf{r}=I-A$$

Unlike Eq. (4), through the present approach, the Dirac delta function is canceled so that this definition of local hardness integrates into a well-defined global hardness, which in fact is equal to $\left(I-A\right)$, which is the quantity identified with the intuitive concept developed by Pearson. It is important to note that the expression in Eq. (15) was first proposed intuitively by Meneses et al. [49, 50], and it was later derived through an approach based on the chain rule for functional derivatives [33, 34].

In order to obtain the chemical potential and hardness kernels, we need to consider first the development of the Fukui kernel [39]. As in the case of the chemical potential, the Fukui function may be expressed as the ratio of the thermal fluctuations between $\langle N\rangle$ and $\langle \rho \left(\mathbf{r}\right)\rangle$ and the thermal fluctuations of $\langle N\rangle$, that is

17 $$f\left(\mathbf{r}\right)=\frac{\langle \rho \left(\mathbf{r}\right)N\rangle -\langle \rho \left(\mathbf{r}\right)\rangle \langle N\rangle }{\langle NN\rangle -\langle N\rangle \langle N\rangle } ,$$

so that using Eq. (9) one can define a Fukui kernel through the expression

18 $$f\left(\mathbf{r},\mathbf{r}\mathbf{^{\prime}}\right)=\frac{\langle \rho \left(\mathbf{r}\right)\rho \left(\mathbf{r}\mathbf{^{\prime}}\right)\rangle -\langle \rho \left(\mathbf{r}\right)\rangle \langle \rho \left(\mathbf{r}\mathbf{^{\prime}}\right)\rangle }{\langle NN\rangle -\langle N\rangle \langle N\rangle } ,$$

that satisfies the relationship

19 $$f\left(\mathbf{r}\right)=\int f\left(\mathbf{r},\mathbf{r}\mathbf{^{\prime}}\right) d\mathbf{r}\mathbf{^{\prime}}.$$

Again, through the three-ground-state ensemble, one finds that in the limit when the temperature goes to zero and $\omega$ is equal to zero

20 $$f\left(\mathbf{r},\mathbf{r}\mathbf{^{\prime}}\right)=\left({f}^{-}\left(\mathbf{r}\right){f}^{-}\left(\mathbf{r}\mathbf{^{\prime}}\right)+{{f}^{+}\left(\mathbf{r}\right)f}^{+}\left(\mathbf{r}\mathbf{^{\prime}}\right)\right)/2$$

Considering Eq. (19), one can see that in order to recover Eq. (5), through the integration over $\mathbf{r}{\prime}$ in Eq. (20), requires that

21 $${f}^{-}\left(\mathbf{r}\right)=\int {f}^{-}\left(\mathbf{r},\mathbf{r}\mathbf{^{\prime}}\right) d{\mathbf{r}}^{\mathbf{^{\prime}}}=\int {f}^{-}\left(\mathbf{r}\right) {f}^{-}\left(\mathbf{r}\mathbf{^{\prime}}\right) d{\mathbf{r}}^{\mathbf{^{\prime}}},$$

and that

22 $${f}^{+}\left(\mathbf{r}\right)=\int {f}^{+}\left(\mathbf{r},\mathbf{r}\mathbf{^{\prime}}\right) d{\mathbf{r}}^{\mathbf{^{\prime}}}=\int {f}^{+}\left(\mathbf{r}\right) {f}^{+}\left(\mathbf{r}\mathbf{^{\prime}}\right) d{\mathbf{r}}^{\mathbf{^{\prime}}}.$$

Thus, replacing ${f}^{-}\left(\mathbf{r}\right)$ and ${f}^{+}\left(\mathbf{r}\right)$ in Eqs. (13) and (15) by its expressions in terms of the product given in Eqs. (21) and (22), respectively, and considering in the final expressions only the integrands, one can define the chemical potential kernel as

23 $$\mu \left(\mathbf{r},\mathbf{r}\mathbf{^{\prime}}\right)=-\left(I{f}^{-}\left(\mathbf{r}\right) {f}^{-}\left({\mathbf{r}}^{\mathbf{^{\prime}}}\right)+A{f}^{+}\left(\mathbf{r}\right){f}^{+}\left({\mathbf{r}}^{\mathbf{^{\prime}}}\right)\right)/2 ,$$

and the hardness kernel as

24 $$\eta \left(\mathbf{r},\mathbf{r}\mathbf{^{\prime}}\right)=I{f}^{-}\left(\mathbf{r}\right){f}^{-}\left(\mathbf{r}\mathbf{^{\prime}}\right)-A{f}^{+}\left(\mathbf{r}\right){f}^{+}\left({\mathbf{r}}^{\mathbf{^{\prime}}}\right) .$$

Through the analysis of Eqs. (1), (13), and (23), one can see that

25 $$\mu =\int \mu \left(\mathbf{r}\right) d\mathbf{r}=\iint \mu \left(\mathbf{r},\mathbf{r}\mathbf{^{\prime}}\right)d\mathbf{r}d{\mathbf{r}}^{\mathbf{^{\prime}}},$$

and from Eqs. (16) and (24), one can see that

26 $$\eta =\int \eta \left(\mathbf{r}\right) d\mathbf{r}=\iint \eta \left(\mathbf{r},\mathbf{r}\mathbf{^{\prime}}\right)d\mathbf{r}d{\mathbf{r}}^{\mathbf{^{\prime}}}=I-A.$$

It is important to emphasize that in the grand canonical ensemble approach, the derivatives for $\omega =0$ are determined first for the temperature-dependent expression, and the resulting equation is evaluated at $T=0$. Thus, since an important element of this procedure comes from the fact that the effect of temperature is to smoothen the behavior of $\langle E\rangle$ and $\langle \rho \left(\mathbf{r}\right)\rangle$ with respect to $\langle N\rangle$, the results reported for $\omega =0$, in all the cases considered in this work, are exact. That is, through this approach, one does not need to invoke the average of the expressions for $\omega <0$ and $\omega >0$ as an approximation for $\omega =0$, which is the procedure that needs to be followed when one calculates the derivatives with respect to $\langle N\rangle$ through the zero-temperature expression, since in such cases, the left and right derivatives are different because of the discontinuities.

Now, the expressions derived with a methodology based on a zero-temperature CDFT description that makes use of the chain rule for functional derivatives, combined with a smooth quadratic interpolation of the energy and of the electronic density among the systems with ${N}_{0}-1$, ${N}_{0}$, and ${N}_{0}+1$ electrons in their ground state, to calculate the first and the second derivatives of $\langle E\rangle$ and $\langle \rho \left(\mathbf{r}\right)\rangle$ with respect to $\langle N\rangle$ lead to the same expressions obtained in the cases of the local hardness and the hardness kernel and to slightly different expressions for the local chemical potential and the chemical potential kernel, because in these two cases, through this approach, one ends up with a local chemical potential that it is given by the product of $\mu$ as given in Eq. (1), times $f\left(\mathbf{r}\right)$ as given in Eq. (5), and similarly for the chemical potential kernel where the $f\left(\mathbf{r}\mathbf{^{\prime}}\right)$ will also be given by Eq. (5). However, in the case of the grand canonical ensemble approach considered in this work, the derivation of the local chemical potential through Eq. (11) does not imply that the local chemical potential is given by the product of the global chemical potential times the Fukui function so that it leads to an expression that implies as if it is directly the product of the global chemical potential times the Fukui function, the one that is evaluated through the average of the left and right derivatives of the product, although it is important to note that in the grand canonical ensemble formulation, the expression at $\omega =0$ is not an approximation, but it is an exact result. Additionally, in the case of the evaluation of the product of the global chemical potential times the Fukui function with Eqs. (1) and (5), which are obtained through the chain rule approach, one may be led to terms that mix the propensity to donate charge, measured by $I$ or ${f}^{-}\left(\mathbf{r}\right)$ with the propensity to accept the charge, measured by $A$ or ${f}^{+}\left(\mathbf{r}\right)$, because the indicated product leads to the appearance of terms of the form $I{f}^{+}\left(\mathbf{r}\right)$ and $A{f}^{-}\left(\mathbf{r}\right)$, which mixes the two propensities. For all these considerations, we believe that the expressions for the local chemical potential and the chemical potential kernel derived in this work, through the grand canonical ensemble, are the appropriate ones to describe these two properties, in addition to the fact that the numerical difference between the two formulations turns out to be small.

Finally, an important aspect of the local and non-local chemical potential and hardness expressions lies in the fact that in all cases, they are composed of the product of the first ionization potential or the electron affinity, which are global reactivity descriptors, times the directional Fukui functions which are local reactivity descriptors. Thus, one can analyze analogous sites or bonds of a series of molecules characterized by similar directional Fukui functions values, in different chemical environments characterized through the values of the global properties.

## Results and discussion

In order to determine the global and local quantities that are present in the definitions of the local chemical potential, the local hardness, and the chemical potential and hardness kernels, one can make use of the frontier orbitals to determine their values, that is, for the global properties [51]

27 $$I\approx -{\varepsilon }_{H} ,$$

and

28 $$A\approx -{\varepsilon }_{L} ,$$

where ${\varepsilon }_{H}$ and ${\varepsilon }_{L}$ are the eigenvalues of the highest (H) occupied and lowest (L) unoccupied molecular orbitals, while for the local properties, one first approximates ${f}^{-}\left(\mathbf{r}\right)$ and ${f}^{+}\left(\mathbf{r}\right)$ with the densities of the frontier orbitals, ${\rho }_{H}\left(\mathbf{r}\right)$ and ${\rho }_{L}\left(\mathbf{r}\right)$, respectively [18, 20], and then, since these are functions of the position in space, it is more convenient to replace them with the condensed-to-atom Fukui functions [52, 53] that may be evaluated through a Hirshfeld population analysis [54], with the distribution function ${w}_{\text{Hirsh}}\left(\mathbf{r}\right)$, so that

29 $${{f}_{k}^{-}\approx f}_{k}^{H}=\int {w}_{\text{Hirsh}}\left(\mathbf{r}\right) {\rho }_{H}\left(\mathbf{r}\right) d\mathbf{r}\boldsymbol{ }\boldsymbol{ }\boldsymbol{ }\boldsymbol{ }\boldsymbol{ },$$

and

30 $${{f}_{k}^{+}\approx f}_{k}^{L}=\int {w}_{\text{Hirsh}}\left(\mathbf{r}\right) {\rho }_{L}\left(\mathbf{r}\right) d\mathbf{r}\boldsymbol{ }\boldsymbol{ }\boldsymbol{ }\boldsymbol{ }\boldsymbol{ },$$

where the subindex $k$ indicates the $k$-th atom in the molecule.

Through this procedure, one can see that each atomic site in a molecule is characterized by the condensed value of the local chemical potential and the local hardness and that the product of the condensed values of two atoms describes the chemical potential and the hardness kernels of the bond they form.

The examples considered in the present work, to show the usefulness of the local and non-local counterparts of the chemical potential and the hardness, are the electrophilic addition of hydrogen halides (HX) to several substituted ethenes, the bond enthalpies of chemical reactions where there is a homolytic bond break, the hydration reaction of aldehydes and ketones, and the trans influence in which the lability of the leaving ligand is modified by the ligand opposite to it.

The PBE0 exchange–correlation energy function [55–57] was used in all the electronic structure calculations required to obtain the quantities associated with the frontier orbitals in Eqs. (27)–(30). For all the molecules analyzed, the geometry optimization was done with a modified version of the NWChem [58] program, while the condensed Fukui functions ${f}_{k}^{H}$ and ${f}_{k}^{L}$ were determined with a developer version of the deMon2k program [59] in which the electronic structure of each molecule was calculated with the same optimized geometry, functional, and basis set used in the NWChem calculation, together with a GEN-A2* auxiliary basis [60], to perform a Hirshfeld population analysis that it is, basically, equal the one obtained through NWChem, but that additionally determines the values of ${f}_{k}^{H}$ and ${f}_{k}^{L}$.

**Fig. 1:**
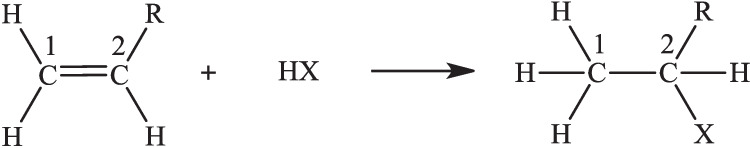
Electrophilic addition reaction of hydrogen halides to substituted ethenes with Markovnikov’s rule orientation

Thus, for the case of the electrophilic addition, we present in Fig. 1 the reaction associated with this process, where we have considered that the product corresponds to the case when the Markovnikov rule is followed [61] so that the hydrogen in HX (X is an halide atom) is the one that binds to the carbon atom of the double bond that has more hydrogens bonded to it, that is, C1 in Fig. 1.

For this process, given the relevance of C1 and the double bond between C1 and C2, one may consider that the local quantities analyzed in this work, evaluated at C1, may provide information about the factors that characterize this carbon atom with respect to the activation energy of the reaction. On the other hand, for the role of the double bond, one may consider the non-local quantities studied in this work, with respect also to this property.

**Fig. 2:**
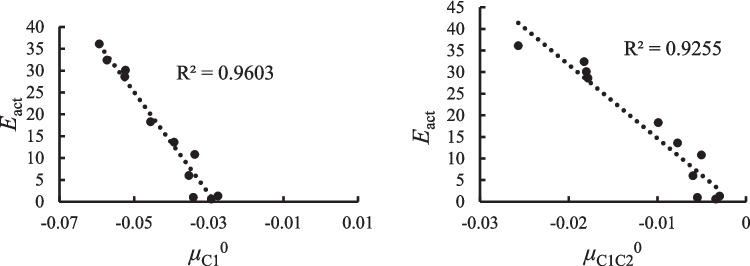
Correlation profiles for the activation energy in kcal/mol with the local chemical potential (left panel) and with the chemical potential kernel (right panel), both in hartrees

**Fig. 3:**
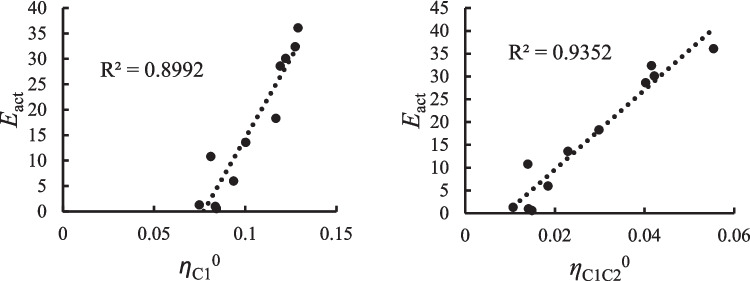
Correlation profiles for the activation energy in kcal/mol with the local hardness (left panel) and with the hardness kernel (right panel), both in hartrees

The substituents R considered were NH_2_, OH, CH_3_, OCH_3_, NHCH_3_, NHOH, N(CH_3_)_2_, NHNH_2_, CH_2_CH_3_, F, and H. The calculations of the electronic structure of the different species were done using the 6-311G** basis set [62, 63]. The analysis presented is centered on the activation energy calculated for these systems [50]. In Table S1 of the supplementary information, we provide the values of these activation energies, together with the data required for the calculation of the reactivity indexes considered. In the left panel of Fig. 2, we present the behavior of the activation energy of the reaction described in Fig. 1 with respect to the local chemical potential evaluated at C1. One can see that there is a good correlation between these two quantities, and the negative slope indicates that the greater the chemical potential, the lower the activation energy, which is the expected trend in an electrophilic addition in which C1 donates charge to the H of HX, because a greater chemical potential favors the donation process and consequently lowers the activation energy. In the right panel of Fig. 2, one can see the same information as the one presented in the left panel, but in this case, the behavior is analyzed with respect to the chemical potential kernel, which shows a slightly lower correlation, but with similar trends. In Fig. 3, we present a similar analysis for the local hardness and the hardness kernel. In this case, the slope in both plots becomes positive, which implies, as it should, that the harder the site at C1 or the bond between C1 and C2, the greater the activation energy.

The plots in Figs. 2 and 3 underscore the relevance of C1 (local) and the double bond between C1 and C2 (kernel) in this electrophilic addition and show that through these four reactivity indexes, one can analyze the reactivity of these systems. Interestingly, these results suggest that both the acidity and hardness of the reactants play crucial roles in describing the observed patterns for this family of reactions. Acidity is primarily influenced by the C1 atom of the substituted ethene, while the hard-soft interactions predominantly occur through the C1 = C2 double bond.

**Fig. 4:**
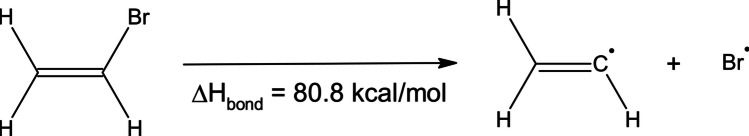
Example of the experimental homolytic bond break carried out to determine bond enthalpies

In the case of the bond enthalpies of chemical reactions where there is a homolytic bond break, we considered two groups of molecules which are given in Table S2 of the supplementary information, together with the experimental information that was taken from Ref. [64] and the data required for the calculation of the reactivity indexes considered. The calculations in this case were done with a Def2-TZVP basis set [65]. In Fig. 4, we present, as an example, the experimental reaction done to determine the bond enthalpies for one of the systems considered.

**Fig. 5:**
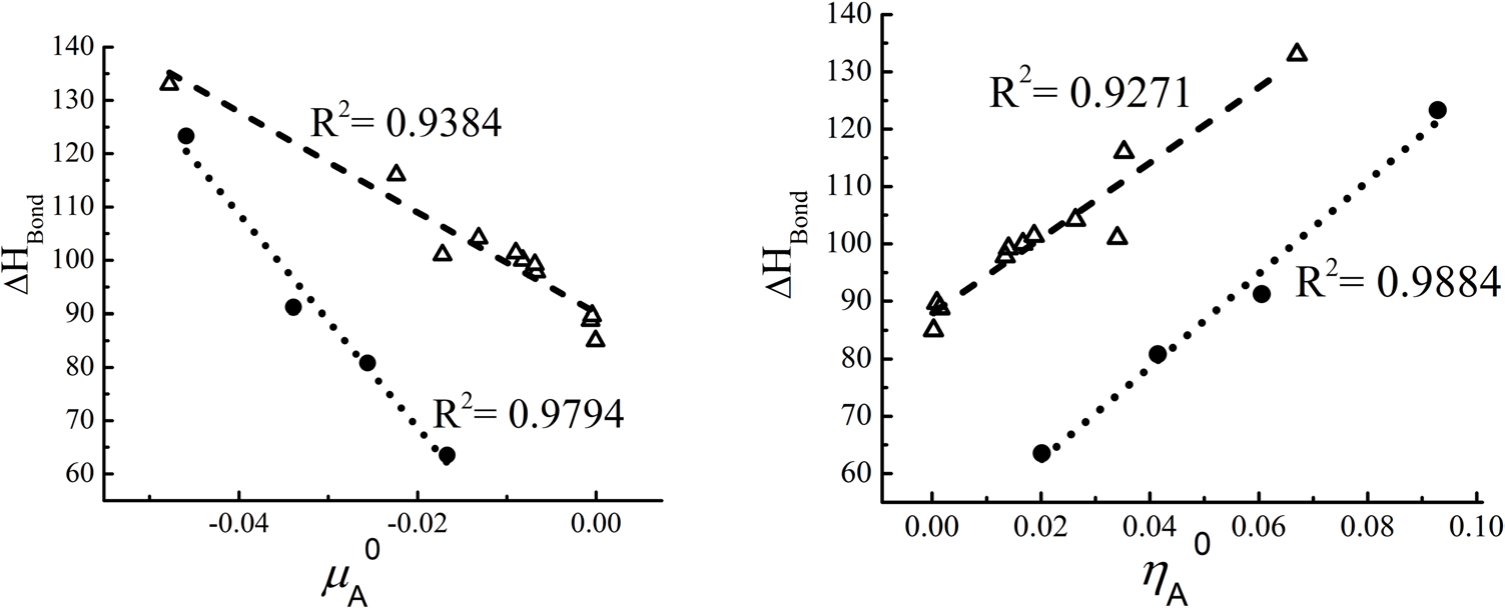
Correlation profiles for the bond enthalpy in kcal/mol with the local chemical potential (left panel) and with the local hardness at atom A (right panel), both in hartrees

In this case, the local chemical potential and the local hardness of the atom defined as A correlate rather well with the experimental bond enthalpy, as may be seen in Fig. 5. In Table S3 of the supplementary information, we specify for each of the molecules considered which is the atom A and which is the atom X. The results obtained indicate that the greater the local chemical potential at this site, the lower the bond enthalpy, and since a greater chemical potential indicates a larger propensity to donate charge, one can see in the left panel of Fig. 5 that this facilitates the bond breaking. On the other hand, in the right panel of Fig. 5, one can observe that the greater the local hardness also of this site, the greater the bond enthalpy, as could be expected. It is important to note that precisely in this case, the chain rule approach led to a good correlation with the kernel of chemical potential of the bond that is being broken, while in the grand canonical ensemble approach developed in the present work, it does not lead to a good correlation. A plausible explanation of this behavior lies in the fact that the expression given for the Fukui function in Eq. (5), which is the one that is emphasized in the chemical potential kernel obtained through the chain rule, leads to a good correlation because this case corresponds to a reaction that involves radicals. In the case of the grand canonical expressions, the kernel of the chemical potential, Eq. (23), emphasizes the donor–acceptor character of the bond, which is not the main aspect to be considered in the homolytic breaking. However, the good correlation between the bond enthalpy and the local chemical potential and hardness at atom A indicates that these two reactivity indicators at this site constitute the dominant factors to characterize the bond enthalpy of these systems.

**Fig. 6:**
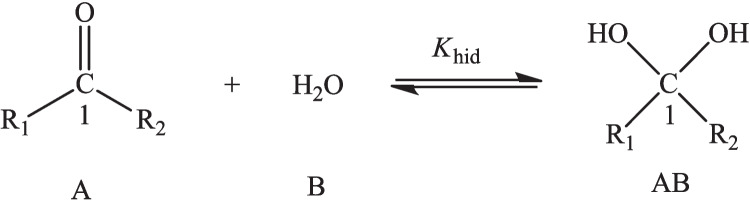
Hydration reaction of aldehydes and ketones for different sets of substituents

A third example we considered is the hydration reaction of aldehydes and ketones in which there is a nucleophilic addition of water over the carbonylic carbon of aldehydes and ketones, with different substituents. The reaction is the one presented in Fig. 6, and the hydration process may be studied through the hydration equilibrium constant, ${K}_{\text{Hyd}}$. The carbon atom where the reaction occurs is denoted as C1. The calculations in this case were done with the 6-311G** basis set [62, 63], and the experimental values for the hydration equilibrium constant were taken from Refs. [66, 67]. These are reported in Table S4 of the supplementary information, together with the data required for the calculation of the reactivity indexes considered.

**Fig. 7:**
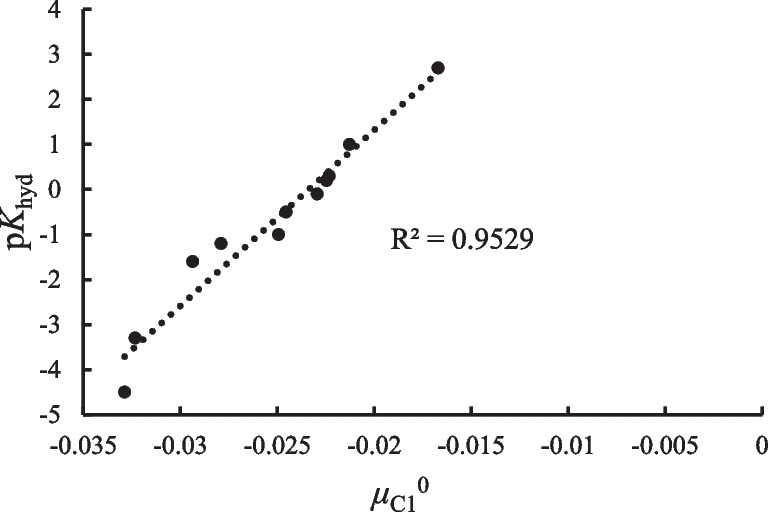
Correlation profiles for the hydration equilibrium constant, of a group of aldehydes and ketones, with the local chemical potential at C1, in hartrees

For this reaction, one can expect that the lower the local chemical potential at this atom, the greater the propensity to accept charge that, according to Fig. 7, leads to a lower $\text{p}{K}_{\text{Hyd}}$, indicating that the equilibrium in this case is displaced toward the products. The local hardness, the chemical potential, and hardness kernels do not correlate with the $\text{p}{K}_{\text{Hyd}}$, leading to the conclusion that the dominant effect in this case corresponds to the charge acceptance capacity of this carbon atom.

**Fig. 8:**
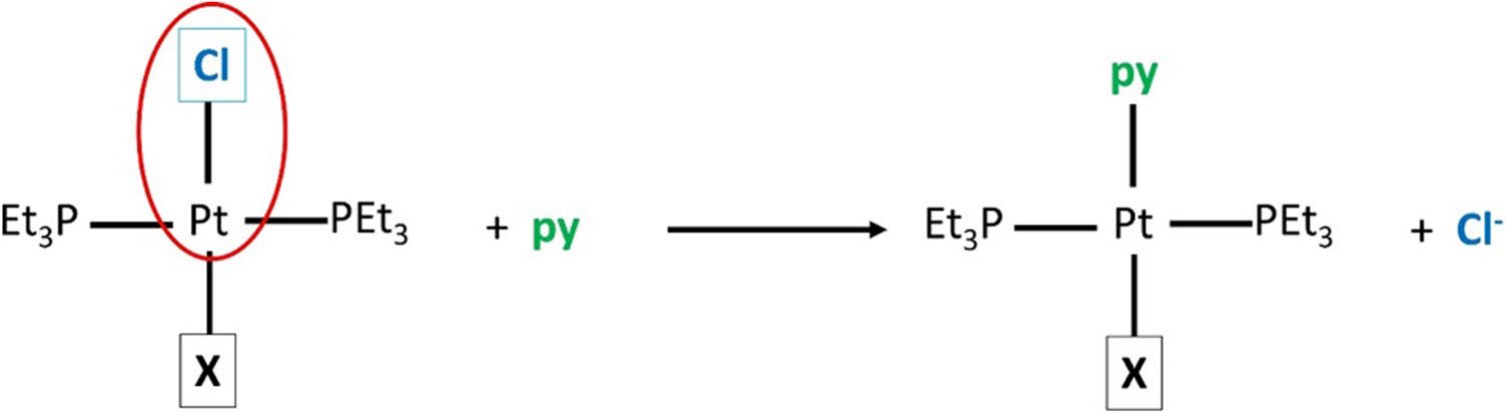
Reaction for the substitution of chloride by pyridine in a square complex of Pt (II)

Finally, the fourth example corresponds to what is known as the trans influence in which the lability of the leaving ligand is modified by the ligand opposite to it [68]. This statement may be verified through the reaction rate constant of the substitution of a given ligand in similar environments, where the trans ligand of the leaving group is changed. In this context, Basolo et al. reported the reaction rate constant for the substitution of chloride by pyridine in a square complex of Pt (II) [69, 70], to prove the trans influence (Fig. 8). The order of the rate constants found for ligands located at the trans position to Cl and that have a Pt-Cl bond was

$$\text{Me }>\text{ pCl}-\text{Ph }=\text{ Ph }>\text{ pMeO}-\text{Ph }>\text{ biPh}$$

It is expected that the ligands with greater polarizabilities will have a greater trans influence and will show greater rates of reaction; simultaneously, the ligands with large retrodonation will also show greater rates of reaction [68, 69]. In the order observed by Basolo et al., it is difficult to establish a tendency with the polarizability or the retrodonation capacity of the trans ligands. However, it is clear that there is a change in the rate due to the presence of different ligands at the trans position, which can be confirmed through the experimental values reported in Table S5 of the supporting information, where we also provide the data required to evaluate the reactivity indexes considered.

**Fig. 9:**
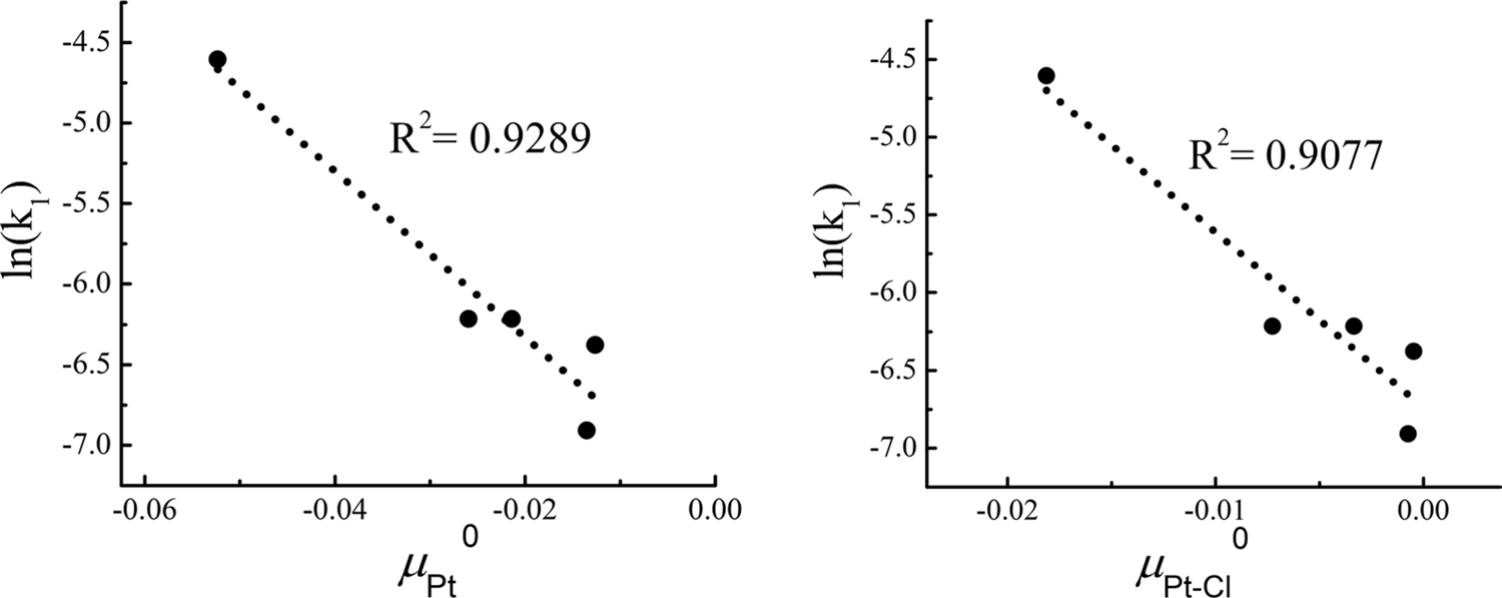
Correlation profiles for the rate of the reaction described in Fig. 8, with the local chemical potential at the Pt atom (left panel) and with the chemical potential kernel of the Pt-Cl bond (right panel), both in hartrees

**Fig. 10:**
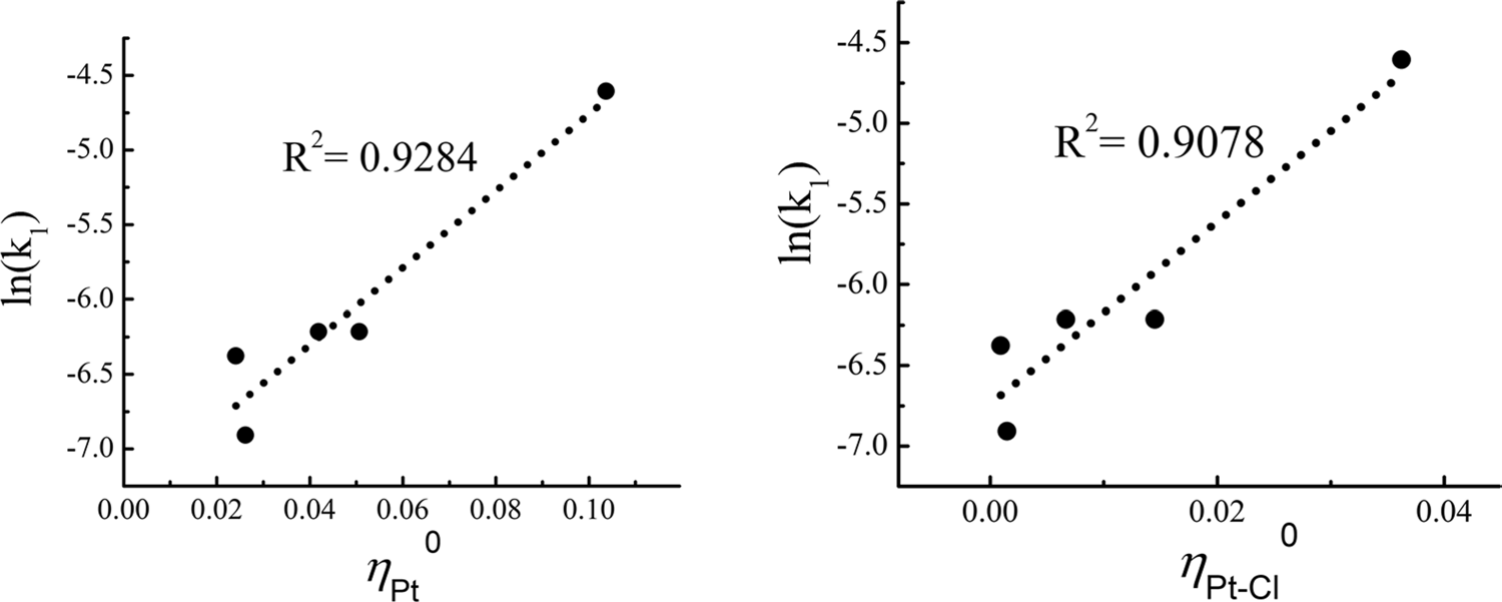
Correlation profiles for the rate of the reaction described in Fig. 8, with the local hardness at the Pt atom (left panel) and with the hardness kernel of the Pt-Cl bond (right panel), both in hartrees

In the present work, we analyzed the influence of these five groups, through the local chemical potential and hardness and the chemical potential and hardness kernels. The calculations were done with a Def2-TZVP basis set [65] and a pseudopotential (Def2-ECP) for platinum [71, 72]. Thus, in the left panel of Fig. 9, we present the behavior of the rate of the reaction described in Fig. 8, $\text{ln}{k}_{1}$, with respect to the local chemical potential evaluated at the Pt atom. One can see that there is a good correlation between these two quantities. The negative slope implies that the greater the chemical potential, the lower the rate of reaction, that is, when the propensity to donate charge increases the rate of reaction diminishes. In the right panel of this Fig. 9, we present the behavior of $\text{ln}{k}_{1}$ with respect to the chemical potential kernel of the Pt-Cl bond that corresponds to the bond associated with the leaving group. One can see that in this case, there is a similar correlation and also a negative slope that indicates a similar trend to the one obtained for the local indicator. Finally, Fig. 10 shows the behavior of the rate constant with respect to the local hardness and the hardness kernel. For these two reactivity indexes, the slope becomes positive, which implies that both, a harder Pt and a harder Pt-Cl bond, increase the reaction rate.

The plots in Figs. 9 and 10 highlight the critical role of both the local chemical potential and hardness at the Pt atom, as well as the chemical potential and hardness kernels associated with the Pt-Cl bond. These observations suggest that while the transition metal is crucial in the substitution step, the reaction itself is more accurately described as a localized process occurring primarily at the Pt-Cl bond instead.

It is important to note that in some of the cases considered here, we were unable to find more of the experimental information required to include a greater number of systems, so the correlations shown in these cases may be uncertain. However, the tendencies shown allow one to perform a qualitative discussion of the chemical implications that derive from the relation of these properties with the reactivity indicators presented in this work.

## Concluding remarks

The development of CDFT has shown, through the many examples reported in the literature, that, indeed, the global chemical potential and hardness that characterize the molecule as a whole, together with the directional Fukui functions that provide information about the characteristics of the different sites in a molecule, are rather important quantities to describe the intrinsic chemical reactivity of a given species. That is, it may be considered that these reactivity indexes transform the relevant, but complex, information contained in the wavefunction obtained through an electronic structure calculation, into chemically meaningful quantities, that allow one to analyze the behavior of thermodynamic and kinetic properties with respect to them.

In the present work, we have revisited the grand ensemble approach to derive the local counterpart of the global chemical potential and hardness and the non-local counterpart of the directional Fukui functions and have combined them to derive the non-local counterparts associated also with the two global properties. Thus, through this approach, one may obtain additional information about the sites (local) and bonds (non-local) that make the greatest contribution to the global value.

A relevant aspect of the local and non-local chemical potential and hardness expressions lies in the fact that in all cases they are composed of terms that contain the product of the first ionization potential and the directional Fukui function ${f}^{-}$ evaluated at $\mathbf{r}$ or $\mathbf{r}\mathbf{^{\prime}}$, both related to charge donation, the first one at the global and the second one at the local levels, and terms that contain the electron affinity and the directional Fukui function ${f}^{+}$ evaluated at $\mathbf{r}$ or $\mathbf{r}\mathbf{^{\prime}}$, both related to charge acceptance, also the first one at the global and the second one at the local levels. Thus, in the grand canonical ensemble approach, there are no crossed terms of the product of one of them related to donation and another related to acceptance. However, as in the case of the chain rule approach, an important contribution of these local and non-local indexes lies in the fact that one can analyze analogous sites or bonds of a series of molecules characterized by similar directional Fukui function values, in different chemical environments characterized through the values of the global properties.

An important conclusion which can be extracted from the cases analyzed is, whether there is a good correlation or no correlation of these indexes with a property, both situations provide information about the reactivity aspects that may be associated with the behavior observed. Therefore, the development of the local and non-local components of a global descriptor leads to a more complete and integrated view of chemical reactivity through conceptual density functional theory.

## Supplementary Information

Below is the link to the electronic supplementary material.

Supplementary file1 (PDF 319 KB)

## Data Availability

Data is provided within the manuscript and in the supplementary information file.
