# Supplementary material for: Local and non-local chemical potential and hardness: a grand canonical ensemble approach
Source: J Mol Model. 2025 Feb 18;31(3):90. doi: 10.1007/s00894-025-06311-0 (PMC11836152; doi:10.1007/s00894-025-06311-0)
Supplement: Supplementary file 1 — Supplementary file1 (PDF 319 KB) [file 894_2025_6311_MOESM1_ESM.pdf]

Supplementary Information

Local and non-local chemical potential and hardness: A grand canonical ensemble approach

Paulino Zerón,[1*] Maurizio A. Pantoja-Hernández,[1*] Marco Franco-Pérez[2] and José L. Gázquez[1*]

[1]*Universidad Autónoma Metropolitana-Iztapalapa, Departamento de Química, Av. San Rafael Atlixco 186, Ciudad de Mexico 09340, Mexico*

[2]*Universidad Nacional Autónoma de México, Cd. Universitaria, Facultad de Química, Ciudad de Mexico 04510, Mexico*

This Supplementary Information contains the values of the quantities required to evaluate the global, local and non-local (kernel) chemical potential and hardness and the experimental information for the electrophilic addition of hydrogen halides (HX) to several substituted ethenes, the bond enthalpies of chemical reactions where there is an homolytic bond break, the hydration reaction of aldehydes and ketones, and the trans influence in which the lability of the leaving ligand is modified by the ligand opposite to it

Tables

S1. Substituted ethenes Page 2

S2. Bond enthalpies Page 3

S3. Identification of atoms A and X for bond enthalpies Page 4

S4. Hydration of aldehydes and ketones Page 5

S5. Trans influence Page 6

Table S1. Substituted ethenes, activation energy, ionization potential, electron affinity and Fukui functions for the HOMO (f^H) and LUMO (f^L) of the atoms involved in the local quantities and in the kernels.

Substituent	Eact (kcal/mol)	I (Hartrees)	A (Hartrees)	${f_{C1}}^{H}$	${f_{C1}}^{L}$	${f_{C2}}^{H}$	${f_{C2}}^{L}$
$NHCH_3$	0.6	0.2073	-0.0445	0.3446	0.2886	0.1520	0.3135
$NHNH_2$	1.0	0.2121	-0.0395	0.3580	0.1951	0.1651	0.2046
$N(CH_3)_2$	1.3	0.2040	-0.0427	0.3182	0.2312	0.1282	0.2402
NH_2	6.0	0.2162	-0.0469	0.3799	0.2458	0.1851	0.2811
NHOH	10.8	0.2284	-0.0237	0.3254	0.2878	0.1616	0.2835
OCH_3	13.6	0.2360	-0.0333	0.3790	0.3266	0.2144	0.3441
OH	18.3	0.2467	-0.0383	0.4215	0.3355	0.2387	0.3912
CH_2CH_3	28.6	0.2687	-0.0204	0.4172	0.3488	0.3385	0.3227
CH_3	30.1	0.2683	-0.0238	0.4226	0.3679	0.3449	0.3534
F	32.4	0.2809	-0.0177	0.4307	0.3632	0.3226	0.3937
H	36.1	0.2873	-0.0132	0.4310	0.3887	0.4310	0.3887

Table S2. Bond enthalpies, ionization potential, electron affinity and Fukui functions for the HOMO (f^H) and LUMO (f^L) of the atoms involved in the local quantities and in the kernels. The name of each molecule consists of the fragments that form part of the products after the bond breaking.

Compound	Bond Enthalpy (kcal/mol)	I (Hartrees)	A (Hartrees)	${f_A}^{H}$	${f_A}^{L}$	${f_X}^{H}$	${f_X}^{L}$
Group 1							
Vinyl-Br	80.8	0.2735	0.0148	0.1693	0.3282	0.5091	0.116
Vinyl-Cl	91.2	0.2815	0.0106	0.2280	0.3411	0.3679	0.0859
Vinyl-F	123.3	0.2900	-0.0015	0.3184	0.3617	0.1637	0.0678
Vinyl-I	63.55	0.2611	0.0277	0.1025	0.2379	0.6807	0.5947
Group 2							
Vinyl-CN	133	0.3131	0.0609	0.2597	0.2346	0.106	0.1169
Vinyl-Vinyl	116	0.2505	0.0308	0.1597	0.1543	0.1596	0.1543
Tertbutil-Vinyl	97.8	0.2712	-0.0020	0.0495	0.0054	0.3266	0.0266
Ethyl-Vinyl	100	0.2713	-0.0038	0.061	0.0283	0.3334	0.0751
Methyl-Vinyl	101.4	0.2697	-0.0059	0.0681	0.0637	0.3433	0.2912
Isopropyl-Vinyl	99.2	0.2718	-0.0055	0.0511	0.0245	0.3309	0.0252
H-allyl	88.8	0.2697	-0.0059	0.005	0.0156	0.0681	0.0637
H-benzyl	89.7	0.2560	0.0037	0.0035	0.0107	0.0462	0.0227
H-CH2-allyl	85.0	0.2678	-0.0055	8E-4	0.0104	0.0093	0.0129
Phenyl-methoxy	101.0	0.2320	0.0033	0.1475	0.0557	0.225	0.0071
Phenyl-NH2	104.2	0.2094	0.0016	0.1258	0.0022	0.2742	0.0934

Table S3. Identification of atoms A and X for bond enthalpies

Vinyl-Br<br><br>Grupo 1	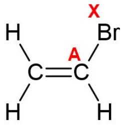	Vinyl-Cl<br><br>Grupo 1	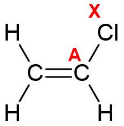
Vinyl-F<br><br>Grupo 1	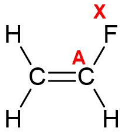	Vinyl-I<br><br>Grupo 1	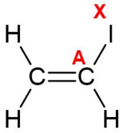
Vinyl-CN<br><br>Grupo 2	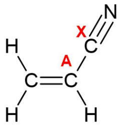	Vinyl-Vinyl<br><br>Grupo 2	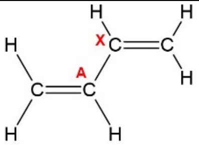
Tertbutil-Vinyl<br><br>Grupo 2	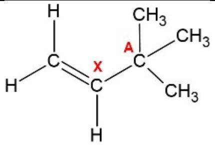	Ethyl-Vinyl<br><br>Grupo 2	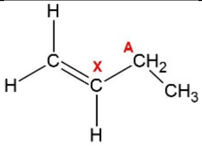
Methyl-Vinyl<br><br>Grupo 2	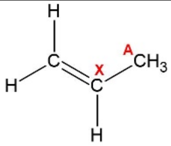	Isopropyl-Vinyl<br><br>Grupo 2	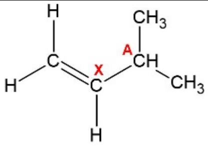
H-allyl<br><br>Grupo 2	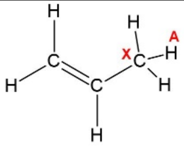	H-benzyl<br><br>Grupo 2	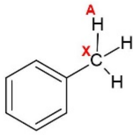
H-CH2-allyl<br><br>Grupo 2	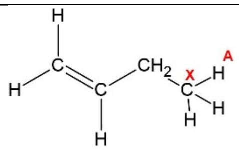	Phenyl-methoxy<br><br>Grupo 2	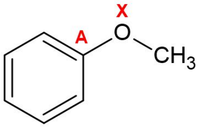
Phenyl-NH2<br><br>Grupo 2	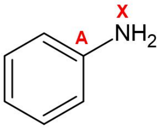		

Table S4. Hydration equilibrium constant, ionization potential, electron affinity and Fukui functions for the HOMO (f^H) and LUMO (f^L) of the atoms involved in the local quantities and in the kernels.

R_1	R_2	pK_{hyd}	I (Hartrees)	A (Hartrees)	${f_{C1}}^{H}$	${f_{C1}}^{L}$	${f_O}^{H}$	${f_O}^{L}$
CCl_3	H	-4.5	0.3162	0.0741	0.1268	0.3462	0.5015	0.2908
H	H	-3.3	0.2814	0.0371	0.1633	0.5053	0.6227	0.3609
CH_2Cl	H	-1.6	0.2926	0.0472	0.133	0.4195	0.5487	0.3211
$CHClCH_2CH_3$	H	-1.2	0.2874	0.0457	0.1319	0.3919	0.5182	0.3067
CH_2Cl	CH_2Cl	-1.0	0.2979	0.0409	0.1145	0.3857	0.4981	0.3193
$CHCl_2$	CH_3	-0.5	0.2931	0.0555	0.1103	0.3025	0.5220	0.2587
CH_3	H	-0.1	0.2708	0.0174	0.1405	0.4521	0.6160	0.3424
CH_2CH_3	H	0.2	0.2692	0.0149	0.1419	0.4512	0.5921	0.3379
$CH_2CH_2CH_3$	H	0.3	0.2681	0.0147	0.1418	0.4509	0.5853	0.3368
CH_2Cl	CH_3	1.0	0.2794	0.0304	0.1103	0.3861	0.5398	0.3005
CH_3	CH_3	2.7	0.2617	0.0075	0.1163	0.3945	0.6179	0.3165

Table S5. Rate of reaction, ionization potential, electron affinity and Fukui functions for the HOMO (f^H) and LUMO (f^L) of the atoms involved in the local quantities and in the kernels.

Compound	$\ln k_1$	I (Hartrees)	A (Hartrees)	${f_{Pt}}^H$	${f_{Pt}}^L$	${f_{Cl}}^H$	${f_{Cl}}^L$
$[Pt(PEt_3)_2($**Me**$)Cl]$	-4.605	0.2246	0.0080	0.4638	0.0633	0.3480	0.0290
$[Pt(PEt_3)_2($**pCl-Ph**$)Cl]$	-6.215	0.2314	0.0108	0.1829	0.0422	0.1577	0.0066
$[Pt(PEt_3)_2($**Ph**$)Cl]$	-6.215	0.2299	0.0079	0.2230	0.0782	0.2830	0.0298
$[Pt(PEt_3)_2($**pMeO-Ph**$)Cl]$	-6.378	0.2124	0.0085	0.1162	0.0656	0.0378	0.0186
$[Pt(PEt_3)_2($**biPh**$)Cl]$	-6.908	0.2207	0.0235	0.1206	0.0180	0.0553	0.0006
